# The Allelopathic Effects of *Trewia nudiflora* Leaf Extracts and Its Identified Substances

**DOI:** 10.3390/plants12061375

**Published:** 2023-03-20

**Authors:** Mst. Rokeya Khatun, Shunya Tojo, Toshiaki Teruya, Hisashi Kato-Noguchi

**Affiliations:** 1 Department of Applied Biological Science, Faculty of Agriculture, Kagawa University, Miki 761-0795, Kagawa, Japan; 2 The United Graduate School of Agricultural Sciences, Ehime University, 3-5-7 Tarumi, Matsuyama 790-8566, Ehime, Japan; 3 Department of Entomology, Faculty of Agriculture, Bangladesh Agricultural University, Mymensingh 2202, Bangladesh; 4 Graduate School of Engineering and Science, University of the Ryukyus, 1 Senbaru, Nishihara 903-0213, Okinawa, Japan; 5 Faculty of Education, University of the Ryukyus, 1 Senbaru, Nishihara 903-0213, Okinawa, Japan

**Keywords:** *Trewia nudiflora*, allelopathic substances, loliolide, 6,7,8-trimethoxycoumarin, weed control

## Abstract

*Trewia nudiflora* Linn. is a woody plant of the Euphorbiaceae family. It is well known for its use as a folk remedy, but its potential for phytotoxicity has not been explored. Therefore, this study investigated the allelopathic potential and the allelopathic substances in *T. nudiflora* leaves. The aqueous methanol extract of *T. nudiflora* was found to have a toxic effect on the plants used in the experiment. The shoot and root development of lettuce (*Lactuca sativa* L.) and foxtail fescue (*Vulpia myuros* L.) were significantly (*p* ≤ 0.05) reduced by the *T. nudiflora* extracts. The growth inhibition by the *T. nudiflora* extracts was proportional to the extract concentration and varied with the test plant species. The chromatographic separation of the extracts resulted in the isolation of two substances, identified as loliolide and 6,7,8-trimethoxycoumarin based on their respective spectral analyses. Both substances significantly inhibited lettuce growth at a concentration of 0.01 mM. To inhibit 50% of the growth of the lettuce, the required concentration of loliolide was 0.043 to 0.128 mM, while that of 6,7,8-trimethoxycoumarin was 0.028 to 0.032 mM. Comparing these values, the lettuce growth was more sensitive to 6,7,8-trimethoxycoumarin than loliolide, suggesting that 6,7,8-trimethoxycoumarin was more effective than loliolide. Therefore, the inhibition of the growth of the lettuce and foxtail fescue suggests that loliolide and 6,7,8-trimethoxycoumarin are responsible for the phytotoxicity of the *T. nudiflora* leaf extracts. Thus, the growth-inhibitory effectiveness of the *T. nudiflora* extracts and the identified loliolide and 6,7,8-trimethoxycoumarin may be used to develop bioherbicides that restrict the growth of weeds.

## 1. Introduction

Weeds diminish crop yields directly and indirectly and increase production expenses, making them the most destructive agricultural pests worldwide. Weed infestation results in a reduction of about 34% of agricultural production [1]. Weeds alone can cause 37% yearly crop production losses depending on the crop and season; the loss of yield might even reach 100% without control measures [2,3,4]. Herbicides are widely used in agricultural production against weeds as an efficient and affordable means of improving the quality and quantity of crops. Herbicides comprise nearly half (47.5%) of the more than four million tons of pesticides used every year across the globe for successful weed management and to boost agricultural output [5,6]. These chemicals, when used irresponsibly, bioaccumulate in the food chain and induce catastrophic effects on ecosystem elements, including humans. The alarming increase in herbicide resistance seen in weed populations across the globe is another cause for concern [7]. These negative outcomes of herbicide application drive the search for an alternative method of weed control that is gentler on the environment and more economical for farmers.

Allelopathy is a natural plant–plant or plant–other microbe interaction in the vicinity that is mediated by allelopathic substances, which are released via root secretions, leaching, volatilization, and plant litter decomposition [8]. Allelopathic plant extracts and the substances they contain have attracted much attention, especially those that have phytotoxic effects on plants. The phytotoxicity of allelopathic substances from various plants could be exploited to prevent the growth of weeds. Biodiversity in plants has offered a rich source of physiologically active components that may provide potential allelopathic substances for bioherbicide production [9]. Bioherbicides are environmentally benign methods of weed control. The diversified structural makeup of plant-derived allelopathic substances contribute to a greater degree of solubility and a shorter half-life than synthetic herbicides [10]. Moreover, these substances have a wide range of modes of action against their targets, stemming from the structural complexity of the substances [11,12]. Therefore, the phytotoxic effects of allelopathic plants and their substances can be used for weed management. Allelopathic additives obtained from several plant extracts and their substances may boost crop output while concurrently reducing weed development. Allelopathic additives could be added as mulch, through intercropping, by planting them as cover crops, or by soil drenching [13]. Different allelopathic substances have been isolated by researchers, such as ferulic acid, p-coumaric acid, catechin, and phloridzin isolated by Al Harun et al. [14] and 1,8-cineole, isomenthol, α-terpineol pinocarvone, and globulol isolated by Zhang et al. [15], which showed phytotoxicity against the growth of several test plants. The phytotoxic effects resulted from the interruption of growth processes such as cell division, the rate of elongation and expansion, and the interruption of ATP generation, which is essential for mitosis and is related to the phytotoxicity of allelopathic plant extracts and its substances [16,17,18]. Moreover, allelopathic substances interfere with growth hormones and restrict enzyme functions, which leads to ROS-mediated oxidative stress in plants, which is also responsible for the retardation of plant growth [19,20,21]. Therefore, it is necessary to identify and assess candidate plant species with phytotoxic substances to develop synthetic herbicide substitutes for ecologically friendly crop farming.

Euphorbiaceae is the fifth-largest angiosperm plant family. This family includes more than seven thousand plant species, extending their distribution across the world in both tropical and temperate regions [22,23]. Phytochemicals derived from this family include terpenoids, alkaloids, flavonoids, tannins, lectins, taraxerol, etc., which are diverse in their structures and bioactivities [24,25]. These phytoconstituents have the potential to function as phytotoxic substances that can be employed to curb the growth of weeds. Several species in the Euphorbiaceae family, including *Euphorbia heterophylla* and *Euphorbia guyoniana*, have been shown in previous studies to contain allelopathic constituents that are toxic to a variety of plant species, including *Sorghum bicolor*, *Lactuca sativa*, *Bromus tectorum*, and *Melilotus indica* [26,27]. However, many plants in this family remain unexplored for their phytotoxicity and related substances.

**Figure 1:**
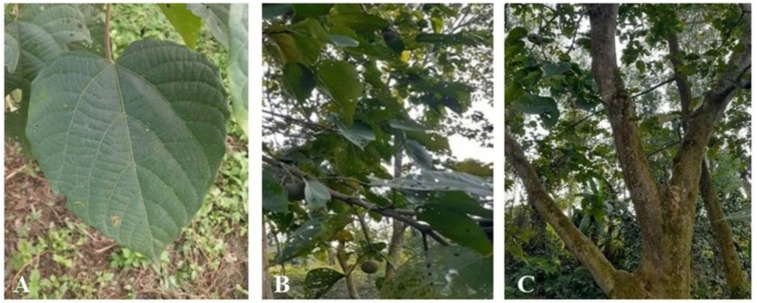
*T. nudiflora* leaf (**A**), branch with fruit (**B**), and trunk (**C**).

*Trewia nudiflora* Linn. is a perennial plant (up to 30 m in height) of the Euphorbiaceae family that is known as false white teak in English and pitali in Bengali. This tropical plant has a broad distribution in countries such as Bangladesh, India, Malaysia, Nepal, and the southern part of China. This plant grows well in areas with high rainfall and well-drained soils. The leaves are petiolate, acuminate, and hairy on both sides (6–21 cm). The leaves are arranged oppositely along the stem and are usually slightly curved. The lamina is elliptical and has smooth edges, with a glossy green upper surface and a lighter green underside. The flowers are dioecious, where the female flowers are green, and the male ones are yellow. The fruit is an indehiscent drupe, round, and has thin, yellowish–green skin (Figure 1) [28,29,30]. *T. nudiflora* is widely known for its therapeutic benefits, in addition to its use as a resource for timber and nutrition. Its fruits play an important role in the diet of the greater one-horned Asian rhino [31]. The leaves, bark, fruit, and roots of *T. nudiflora* are used to treat a variety of illnesses. A decoction made from the bark and leaves of this plant is used for the treatment of flatulence, excessive bile, and sputum, as well as for the relief of edema. The leaves are used to treat wounds. The roots are used to treat stomachache, rheumatism, and gout [28,32]. Multiple investigations have documented the antioxidant [33,34], antitumor [35,36], and antibacterial activity of *T. nudiflora* [37]. The leaves of *T. nudiflora* extracted with ethanol showed hyperactivity and cerebroprotective properties [38]. Esan et al. [39] documented the toxic effect of *T. nudiflora* on the larvae of mosquitoes. Begum [40] showed the cytotoxic effect of an ethanol extract of different parts of *T. nudiflora* along with antibacterial and antioxidant activity. Despite this extensive research, there is no information in the literature about the allelopathic potential of *T. nudiflora*. Moreover, phytochemical profiling of *T. nudiflora* leaf extracts found that this plant included different classes of bioactive compounds, such as flavonoids, phenols, tannins, saponins, etc.; however, their allelopathic actions have not been elucidated [41]. Therefore, the purpose of this research was to reveal the plant’s allelopathic effect as well as to isolate and determine the molecular characterization of the allelopathic substances from the leaf extract of *T. nudiflora*.

## 2. Results

### 2.1. Assessment of T. nudiflora Extracts for Phytotoxic Activity

**Figure 2:**
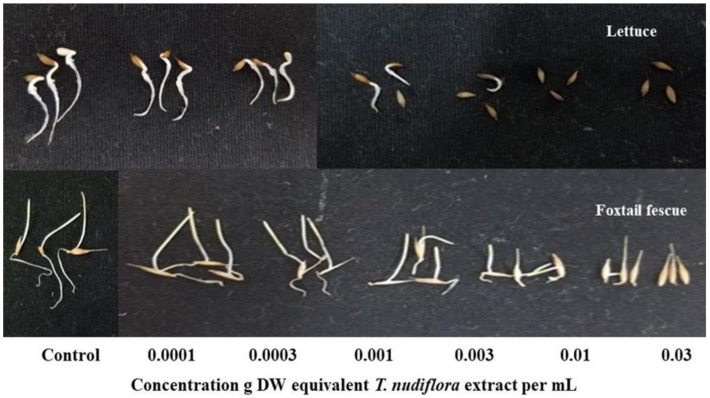
The effect of treatments at six concentrations (control, 0.0001, 0.0003, 0.001, 0.003, 0.01, and 0.03 g DW equivalent *T. nudiflora* extract mL^−1^) on the growth of lettuce and foxtail fescue.

**Figure 3:**
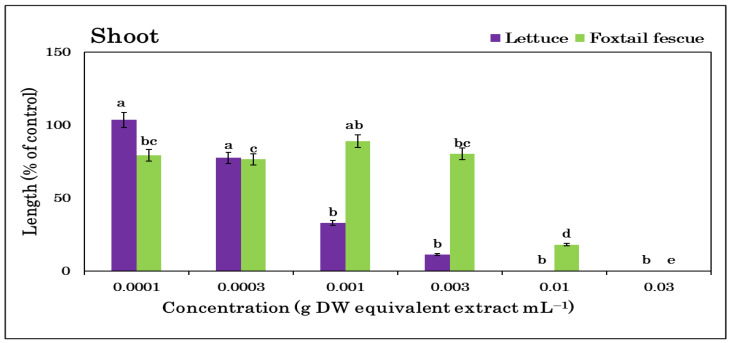
*T. nudiflora* leaf extract treatment at various doses inhibited the shoot growth of the test plants. Mean ± SE of two independent experiments repeated three times. Vertical bars illustrate the standard error of the mean. Different letters stand for differences between the control and *T. nudiflora* treatments (Tukey’s HSD at *p* ≤ 0.05).

**Figure 4:**
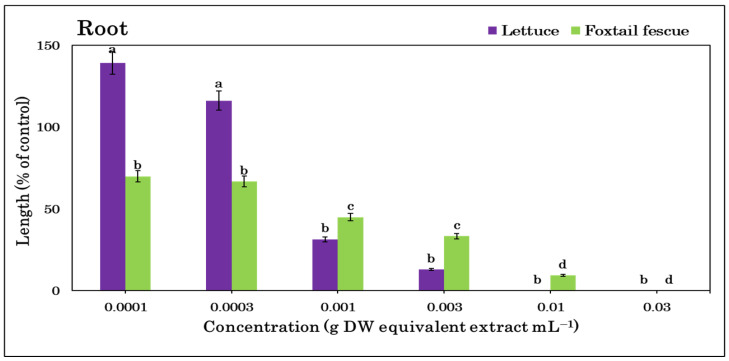
*T. nudiflora* leaf extract treatment at various doses inhibited the root growth of the test plants. Mean ± SE of two independent experiments repeated three times. Vertical bars illustrate the standard error of the mean. Different letters stand for differences between the control and *T. nudiflora* treatments (Tukey’s HSD at *p* ≤ 0.05).

Extracts of *T. nudiflora* were found to be phytotoxic to both lettuce and foxtail fescue (Figure 2) and the phytotoxicity increased with the extract concentrations (Figure 3 and Figure 4). A significant growth reduction was observed in the foxtail fescue at the lowest concentrations (0.0001 and 0.0003 g dry weight (DW)), whereas the lettuce showed no such effects. Except for the foxtail fescue shoots, the concentration of 0.001 g DW equivalent *T. nudiflora* extract mL^−1^ suppressed ≥50% of the shoot and root growth. At a dose of 0.003 g DW equivalent *T. nudiflora* extract mL^−1^, ≥87% of lettuce growth was suppressed, while 19.78 and 66.7% of foxtail shoot and root growth were inhibited, respectively. At 0.01 g DW equivalent *T. nudiflora* extract mL^−1^, the lettuce did not develop at all, and at the same dose, the shoot and root growth of foxtail fescue were suppressed by 82 and 90.63%, respectively. The *T. nudiflora* extracts completely suppressed the shoot and root development of lettuce and foxtail fescue at 0.03 g DW equivalent *T. nudiflora* extract mL^−1^. Extract concentrations of 0.0053–0.0076 g DW equivalent *T. nudiflora* extract mL^−1^ were required to cause a 50% decrease (*I*_50_ values) in the shoot and root development of lettuce and foxtail fescue (Table 1). As seen in Table 1, the foxtail fescue (0.0053–0.0075 g DW) was more sensitive to the *T. nudiflora* extract than lettuce (0.0062–0.0076 g DW).

### 2.2. Characterization of the Active Substances

**Figure 5:**
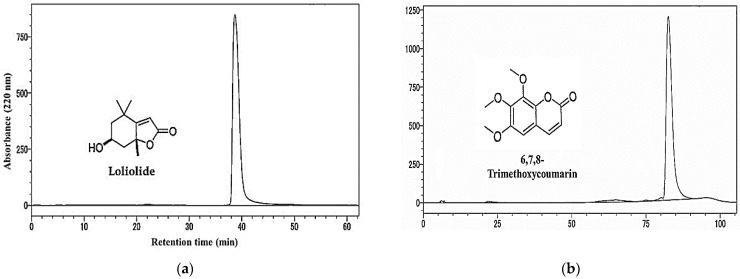
The molecular structure and chromatogram of loliolide (**a**) and 6,7,8-trimethoxycoumarin (**b**) characterized from the *T. nudiflora* leaf extracts.

The molecular formula of substance I was found to be C_11_H_16_O_3_. The ^1^H NMR spectrum of substance I, as measured in the CD_3_OD, showed three methyl proton signals at δ_H_ 1.76 (3H, s), 1.47 (3H, s), and 1.28 (3H, s), one olefinic proton signal at δ_H_ 5.75 (1H, s), one methine proton signal at δ_H_ 4.21 (1H, m), and four methylene proton signals at δ_H_ 2.42 (1H, dt, *J* = 13.8, 2.7), 1.99 (1H, dt, *J* = 14.4, 2.6), 1.75 (1H, dd, *J* = 13.8, 4.1), and 1.53 (1H, dd, *J* = 14.4, 3.7). The ^1^H NMR spectrum of this substance was identified as loliolide and agreed with the reported data of Kim et al. [42] (Figure 5a).

The molecular formula of substance II was found to be C_12_H_12_O_5_. The ^1^H spectrum of substance II, as measured in the CDCl_3_, showed three methyl proton signals at δ_H_ 4.04 (3H, s), 3.99 (3H, s), and 3.90 (3H, s) and three olefinic proton signals at δ_H_ 7.60 (1H, d, *J* = 9.5), 6.66 (1H, s), and 6.34 (1H, d, *J* = 9.5). The ^1^H NMR spectrum of substance II was identified as 6,7,8-trimethoxycoumarin, which agreed with the reported data of Amaral et al. [43] (Figure 5b).

### 2.3. Phytotoxicity of the Two T. nudiflora Extract-Derived Substances

**Figure 6:**
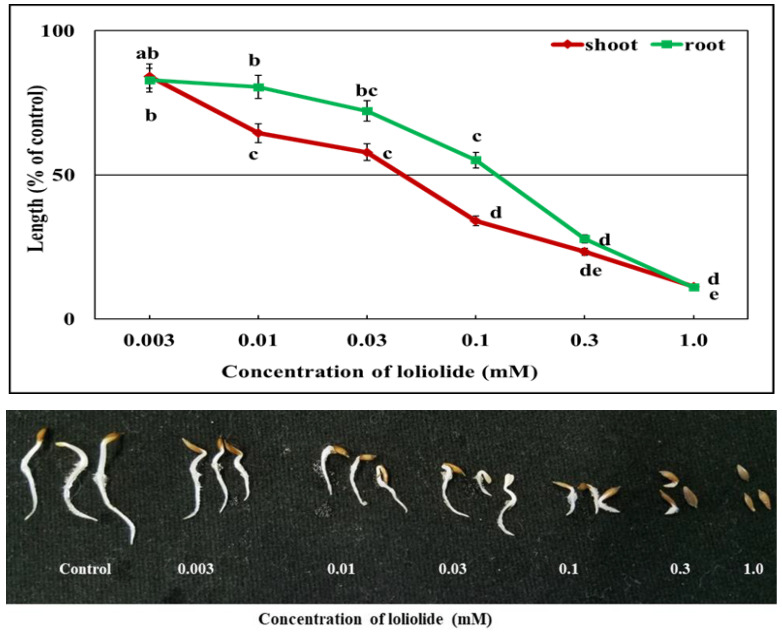
Effects of the loliolide treatment on the growth (shoot and root) of lettuce seedlings. Mean ± SE of each experiment replicated 3 times (*n* = 30). Differences between the control and treatment are represented by different letters (Tukey’s HSD at *p* ≤ 0.05).

**Figure 7:**
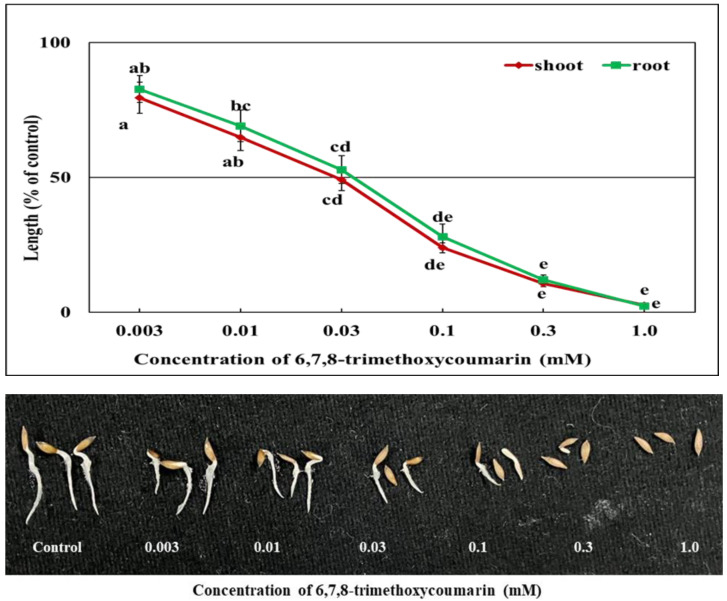
The effects of the 6,7,8-trimethoxycoumarin treatment on the growth (shoot and root) of lettuce seedlings. Mean ± SE of each experiment replicated 3 times (*n* = 30). Differences between the control and treatment are represented by different letters (Tukey’s HSD at *p* ≤ 0.05).

Figure 6 and Figure 7 show that both the loliolide and 6,7,8-trimethoxycoumarin had a significant (*p* ≤ 0.05) effect on the lettuce, which became more prominent with the increase in the concentration. Each substance significantly inhibited the growth of lettuce at a concentration of 0.01 mM. Seedling shoot length decreased by 66 and 76.2% and root length by 44.9 and 72.1% compared with the control when exposed to 0.1 mM loliolide and 6,7,8-trimethoxycoumarin, respectively. At a dose of 0.3 mM, loliolide decreased the lettuce growth by >70%, but 6,7,8-trimethoxycoumarin inhibited it by >85%. At the highest level of loliolide concentration (1.0 mM), the shoot and root length were reduced by 88.8 and 89% compared with the control, respectively; in contrast, 6,7,8-trimethoxycoumarin inhibited the shoot and root length by 97.4 and 97.7% compared with control, respectively. Lettuce shoot and root growth were decreased by 50% with 0.043 and 0.128 mM of loliolide, respectively, which was 1.5 and 4 times higher than 6,7,8-trimethoxycoumarin, indicating that the lettuce was more sensitive to 6,7,8-trimethoxycoumarin than loliolide (Table 2).

## 3. Discussion

The *T. nudiflora* leaf extracts significantly inhibited the growth of lettuce and foxtail fescue (Figure 2), and the extracts had a greater effect on the foxtail fescue and lettuce shoots (Table 1). The leaf extracts of *Leonurus sibiricus* and *Stephania japonica* in a previous study showed similar inhibitory activity against lettuce [44,45]. The phytotoxic effects of *T. nudiflora* were proportional to the extract concentration. An earlier investigation using an extract of *Jatropha curcas* L. from the Euphorbiaceae family showed a comparable dose-responsive inhibitory efficacy [46]. Therefore, this dose- and species-responsive inhibitory activity of *T. nudiflora* suggested its allelopathic potential, which led to the chromatographic separation of the extracts and yielded two phytotoxic substances: loliolide and 6,7,8-trimethoxycoumarin. These substances may be responsible for the phytotoxic effects of *T. nudiflora* observed in the test plants.

Loliolide is a carotenoid metabolite found in many plants, including *Rauvolfia yunnanensis* and *Morus alba*, animals, *Solenopsis invicta*, and sea corals, *Sinularia capillosa* and *Galaxaura filamentosa* [47,48]. This hydroxylactone is well known for its many bioactivities, such as its insect repellent, antimicrobial, and antioxidant activities. [49,50,51]. On the other hand, 6,7,8-trimethoxycoumarin is a naturally occurring simple coumarin identified in plants, mostly in the Apiaceae family. Coumarin is a large class of lactones produced by the shikimic acid pathway [52,53], and 6,7,8-trimethoxycoumarin is 3,4-unsubstituted coumarins [54]. In our study, both substances inhibited the growth of lettuce seedlings (Figure 6 and Figure 7). Our previous research with *Annona reticulata*, from which we extracted loliolide, showed dose- and species-specific growth inhibition of various tested plants [55]. Many studies have identified loliolide as a phytotoxic substance from different plants [56,57] that is toxic to weed (cress and Italian ryegrass) development. The crabgrass root exudates included the substance loliolide. It exhibited phytotoxicity against maize, soybean, and wheat growth as well as interfering with soil microorganism growth [58]. However, there is no such report for 6,7,8-trimethoxycoumarin, although coumarin has been reported to have antioxidant and anti-inflammatory [59,60], anticancer [61], antiparasitic [62], and antimicrobial properties [63]. However, a few coumarins and furanocoumarins isolated from *Zosima absinthifolia* and some of its related families have been shown in some studies to be phytotoxic to test plants [64,65]. The phytotoxic action of coumarins has been described through multiple mechanisms, including interference with oxygen intake and inhibition of cellular mitosis. Compounds belonging to the coumarin family also limit O_2_ uptake, the structural deformation of mitochondria, the inhibition of mitosis in onion root cells [66], the inhibition of ATP production, and oxidative phosphorylation in spinach [67].

To our knowledge, no studies have reported isolating loliolide and 6,7,8-trimethoxycoumarin as phytotoxic substances from the leaf extracts of *T. nudiflora*. The *I*_50_ values of the two substances indicated that the phytotoxicity of the 6,7,8-trimethoxycoumarin against lettuce growth was greater than that of loliolide (Table 2). Dayan et al. [68] speculate that the structural variations of the substances may be the reason for the disparities in effectiveness between them. The scoparone phenyl ring in 6,7,8-trimethoxycoumarin with the methoxy group at the C-5 or C-8 position was reported to enhance some bioactivity, which might be responsible for the greater inhibitory effectiveness of 6,7,8-trimethoxycoumarin [53]. In this research, the *T. nudiflora* extracts and the two substances significantly inhibited the growth of the test plants. Several studies have shown that the integration of plant extracts and their phytoconstituents has herbicidal effects on a variety of weeds due to their diverse structures and mechanisms of action [69]. *Carum carvi* L. extract and its phytotoxic constituents are effective at preventing the establishment of barnyard grass in maize fields [70]. The application of the allelopathic extract of rice straw was found to contain allelopathic substances that inhibited the growth of weeds. Rice straw extract has also been shown to increase crop yields when used as a mulch in rice fields [71]. A study [72] reported that the use of allelopathic rye as a form of mulch could effectively reduce the growth of purslane and redroot pigweed. This was due to the release of phytotoxic benzoxazinoids into the soil by the rye mulch. Many allelopathic substances outperform synthetic herbicides in various ways and are used as key ingredients in bioherbicides that are commercially available. For instance, GreenMatch from *Citrus sinensis* (L.), WeedZap from *Syzygium aromaticum*, and *Cinnamomum verum* are plant-derived commercialized bioherbicides [73,74,75]. *T. nudiflora* is a deciduous plant species, which means it sheds its leaves every year. Effective utilization of its leaves for the reduction in weed growth will decrease the reliance on harsh traditional herbicides. *T. nudiflora* leaves and its substances can be used for weed control through soil additives. The allelopathic extracts of *T. nudiflora* leaves and its phytotoxic substances can be applied as mulch, directly to the soil, or sprayed on the weeds. The leaves of *T. nudiflora* contain allelopathic substances such as loliolide and 6,7,8-trimethoxycoumarin that will be released into the soil as the leaves break down, thus suppressing the growth of weeds. Therefore, *T. nudiflora* extracts as well as the two substances, loliolide and 6,7,8-trimethoxycoumarin, may be exploited for their phytotoxic effectiveness to develop ecofriendly bioherbicides.

## 4. Materials and Methods

### 4.1. T. nudiflora Sample Collection

In April 2020, *T. nudiflora* leaves were collected in Bangladesh (latitude: 24°38′30.12″ N, longitude: 89°39′0.00″ E). The leaves were cleaned under running water, left to air dry in the shade, ground (using a GM 200 Laboratory grinder; Retsch, D-42781 Haan, Germany), and then refrigerated at 2 °C until needed.

### 4.2. Experimental Site

The experimental procedures, both the extraction process of *T. nudiflora* and the bioassay experiment for the extract and the isolated substances from the extract, were carried out in the Plant Biochemistry Laboratory, Department of Applied Biological Science, Kagawa University, Japan. 

### 4.3. Test Plant Species

A growth experiment was conducted using dicot lettuce (*Lactuca sativa* L.) and monocot foxtail fescue (*Vulpia myuros* L.) to investigate the allelopathic action of *T. nudiflora*. Lettuce seeds were purchased from Mikado Kyowa Seed Co., Ltd. (Chiba, Japan), and foxtail fescue seeds were purchased from Snow Brand Seeds Co., Ltd. (Sapporo, Japan). Lettuce is commonly used as an ideal plant for studying allelopathic activity because of its high sensitivity to allelopathic substances. Additionally, it grows rapidly, has a well-established development pattern, and is a widely grown species [76]. In contrast, foxtail fescue is widely recognized as a troublesome weed that invades agricultural fields globally [77].

### 4.4. T. nudiflora Leaf Extraction for the Growth Bioassay

*T. nudiflora* leaf powder (100 g) was extracted two times. First, 1000 mL of 70% (*v*/*v*) aqueous methanol was used to extract the leaf powder for 48 h. The extract was then filtered (using filter paper No. 2; Toyo Roshi Kaisha Ltd., Tokyo, Japan), and the residue was reextracted for 24 h with 1000 mL of methanol and filtered again. A crude extract was obtained by combining the two filtrates and evaporating them at 40 °C (rotary evaporator Model RE 200; Yamato Scientific Co., Ltd., Tokyo, Japan). Different concentrations of *T. nudiflora* (0.0001, 0.0003, 0.001, 0.003, 0.01, and 0.03 g DW equivalent *T. nudiflora* extract mL^−1^) were applied to filter papers (No. 2; Toyo Roshi Kaisha Ltd.) in Petri dishes (28 mm) and dried in a draft chamber. Ten lettuce seeds and ten foxtail fescue seedlings (germinated at 25 °C for 72 h) were placed in the Petri dishes and moistened with 0.6 mL of polyoxyethylene sorbitan monolaurate (0.05% *v*/*v*, Tween 20; Nacalai Tesque, Inc., Kyoto, Japan). The control was moistened with 0.6 mL of 0.05% (*v*/*v*) aqueous Tween 20 solution without an extract solution. The Petri dishes were then transferred to a dark 25 °C growth chamber for 48 h. The shoot and root lengths of the test plants were measured to calculate the growth inhibition percentages using the following equation:

$$\mathrm{Inhibition}\;\left(\%\right)=1-\frac{\mathrm{Length}\;\left(\mathrm{shoot}\;\mathrm{and}\;\mathrm{root}\right)\;\mathrm{with}\;\mathrm{aqueous}\;\mathrm{methanol}\;\mathrm{extract}\;\mathrm{of}\;T.\;\;nudiflora}{\mathrm{Length}\;\left(\mathrm{shoot}\;\mathrm{and}\;\mathrm{root}\right)\mathrm{of}\;\mathrm{control}}\times 100$$

### 4.5. Steps in the Isolation and Purification of the Allelopathic Substances

About 2.90 kg of *T. nudiflora* leaf powder was extracted to obtain an aqueous residue, and the residue was partitioned six times with an equivalent volume of ethyl acetate after being adjusted to pH 7.0 with 1 M phosphate buffer. At each step of the isolation process, the active fraction was determined using a cress bioassay following this methodology [55]. A silica gel column (60 g, silica gel 60, 70–230 mesh; Nacalai Tesque) separated the ethyl acetate fraction eluted with *n*-hexane (20, 30, 40, 50, 60, 70, 80% *v*/*v*; 150 mL each step), ethyl acetate, and 300 mL of methanol. The active fractions (F6) were eluted with 70% ethyl acetate and loaded on a Sephadex LH-20 column (100 g; GE Healthcare, Uppsala, Sweden) with 20, 40, 60, and 80% aqueous methanol (*v*/*v*; 150 mL each step) and methanol (300 mL). The fraction (F2) eluted with 40% aqueous methanol had the most inhibitory activity (more than 95%, data were not presented) and was separated using a C_18_ cartridge (1.2 × 6.5 cm; YMC Co. Ltd., Kyoto, Japan) loaded with 20, 30, 40, 50, 60, 70, 80, and 90% (*v*/*v*) of aqueous methanol (15 mL each step) and methanol (30 mL). The inhibitory activity (more than 80%, data were not presented) was obtained from the elution with 30% aqueous methanol (F2) and was subsequently purified using reverse-phase HPLC with 35% (*v*/*v*) aqueous methanol on a column (500 × 10 mm I.D., S-5 m, 12 nm, YMC Co., Ltd., Kyoto, Japan) at a flow rate of 1.5 mL/min and detected at a wavelength of 220 nm. The peak fractions identified at retention times of 97–103 min (Substance I) and 166–171 min (Substance II) were found to have biological activity and were further purified on a 3 µm-sized column (4.6 I.D. × 250 mm; Inertsil ODS-3, HP 3 µm; GL Sciences Inc., Tokyo, Japan) using reverse-phase HPLC with 30% (*v*/*v*) aqueous methanol at a flow rate of 0.5 mL/min under the same conditions. Two substances were found at retention times of 38–42 and 80–90 min, respectively. HRESIMS was performed on a Thermo Scientific Orbitrap Exploris 240 mass spectrometer, and all NMR spectroscopic data were recorded with an optical rotation of 500 MHz; the substances were identified as colorless oil.

### 4.6. Bioassay of the Identified Substances

The phytotoxicity of the loliolide and 6,7,8-trimethoxycoumarin was examined in relation to the lettuce growth. Solutions of each of the two substances at concentrations of 0.003, 0.01, 0.03, 0.1, 0.3, and 1.0 mM were prepared by dilution with 4 mL of methanol. The bioassay was performed by adding the liquids to filter paper (No. 2), which was then dried in a draft chamber. Ten lettuce seeds were transferred to each Petri dish, along with a control. Tween 20 (0.05% *v*/*v*, 0.6 mL) was used to hydrate the seeds, which were transferred to the growth chamber. After 48 h of treatment, the lettuce growth was measured against the control to determine the percentage of growth inhibition.

### 4.7. Statistical Analysis

The data from a completely randomized design (CRD) were analyzed using one-way ANOVA, and a post-hoc Tukey’s test at *p* = 0.05 was used to identify the statistically significant differences. Every experiment was repeated twice with three biological replications (*n* = 60). The data were analyzed using IBM SPSS version 16.0 [78]. GraphPad Prism 6.0 was used to compute the dose necessary to limit the shoot and root growth of the test plants by 50% (*I*_50_ value).

## 5. Conclusions

The extracts of *T. nudiflora* and their identified substances, loliolide and 6,7,8-trimethoxycoumarin, were phytotoxic to the lettuce and foxtail fescue growth. Both the extracts and the substances significantly inhibited the shoot and root growth of the experimental plants. These findings indicate that the extracts as well as the identified substances can be employed as efficacious, safe, and environmentally friendly substitutes for synthetic herbicides. Further research into the field efficacy of the substances and the mechanisms underlying their anti-growth actions may help in developing biocontrol strategies for weeds in place of synthetic herbicides, thereby assisting in weed management.

## Figures and Tables

**Table 1:** *T. nudiflora* extract concentrations required for 50% shoot and root growth inhibition (*I*_50_ values) of lettuce and foxtail fescue.

Test Plant Species	*I*_50_ Value (g Dry Weight (DW) Equivalent Extract mL^−1^)		
		Shoot	Root
Dicot	Lettuce	0.0062 ^c^	0.0076 ^a^
Monocot	Foxtail fescue	0.0053 ^d^	0.0075 ^b^

Different letters within the same treatment indicate a statistically significant difference (*p* ≤ 0.05) according to the LSD test.

**Table 2:** The *I*_50_ values of loliolide and 6,7,8-trimethoxycoumarin characterized from *T. nudiflora* leaf extracts for lettuce shoot and root growth inhibition.

Test Plant	*I*_50_ Value (mM)		
		Loliolide	6,7,8-Trimethoxycoumarin
Lettuce	Shoot	0.043 ^b^	0.028 ^d^
	Root	0.128 ^a^	0.032 ^c^

Different letters within the same treatment indicate a statistically significant difference (*p* ≤ 0.05) according to the LSD test.

## Data Availability

Not applicable.
